# Dental Hospital, Sydney, New South Wales

**Published:** 1903-01

**Authors:** 


### DENTAL HOSPITAL, SYDNEY, NEW SOUTH WALES.

Sydney, New South Wales, Australia, has the honor of being the first to establish a strictly "Dental Hospital." There have been others in this country and England that work for the poor, but these are in connection with dental colleges.

The official opening of this institution took place on the 17th of October, 1902, His Excellency the Governor Sir Harry Rawson, presiding."

The object of this institution is "To provide *gratuitous* advice and surgical aid and other treatment in all diseases of the mouth and teeth to the *poor and necessitous* who are unable to procure the same at their own expense, and whose cases are suitable for dental treatment, with or without recommendation."

This is to be supported by subscriptions and donations.

A long list of dental surgeons and assistant dental surgeons seem to insure satisfactory work for the poor, and our best wishes go with this experiment, for it is one of the most important efforts to relieve suffering.

The question has been much discussed in this country, and several efforts have been made in this direction, but, so far, with no satisfactory results. The reasons for this are well understood and apparently insurmountable. This work for the poor has, therefore, been left to the college dispensary, and in our large cities has been very satisfactory to those of very limited means. The *very poor* cannot afford the time for prolonged dental operations.

